# A case report of Henoch–Schönlein purpura in the elderly complicated by multisystem involvement

**DOI:** 10.1097/MD.0000000000041163

**Published:** 2025-01-03

**Authors:** Song Na, Lei Zhang, Luxin Kou, Jinquan Xu, Li Gang

**Affiliations:** a Emergency Intensive Care Unit, Affiliated Zhongshan Hospital of Dalian University, Dalian, China.

**Keywords:** elderly, glucocorticoid, Henoch–Schonlein purpura, multisystem involvement, rituximab

## Abstract

**Rationale:**

Henoch–Schönlein purpura (HSP), a vasculitis caused predominantly by immunoglobulin A vasculitis deposition in the blood vessel wall. It frequently affects multiple organs, however, intussusception and cardiac involvement in the elderly is extremely rare. Currently, the diagnosis of patients with atypical HSP is very difficult. Drugs and treatments lack adequate evidence-based medical proof to improve patients’ long-term outcomes. We report a case of an elderly patient with allergic purpura involving the intestines, kidneys and heart, and discuss the diagnosis and treatment of patients with atypical clinical symptoms.

**Patient concerns:**

A 72-year-old woman whose initial symptoms were unusual, and the progression of the disease is irregular.

**Diagnoses:**

The patient with HSP gradually developed intussusception, renal failure and cardiac involvement.

**Interventions and outcomes:**

The patient developed kidney injury and intussusception, we sequentially administered surgical intervention, glucocorticoids, hemofiltration and hemoperfusion, and her condition showed improvement. Unfortunately, by the time cardiac complications develop, the patient’s condition deteriorated rapidly. At last, the patient died.

**Lessons:**

HSP with renal failure, intussusception, and cardiac complication is extremely rare. When patients with atypical HSP, we should always be alerted to changes in their condition. And take into account factors such as the severity of symptoms, patient preferences and complications in order to determine the most suitable therapies.

## 1. Introduction

Henoch–Schönlein purpura (HSP), also known as immunoglobulin A vasculitis (IgA), is founded on the pathology of systemic small-vessel vasculitis and emerges in children and adolescents, more frequently in autumn and winter. HSP is correlated with genetic factors, infections, medications, food and environmental factors, with infections bein0g the predominant cause.^[1]^ HSP can affect multiple systems, with the typical tetrad of signs encompassing palpable purpura, arthritis or arthralgia, abdominal pain and renal involvement. The most common initial manifestations are purpura and joint pain.^[2]^ Moreover, it is classified into cutaneous, abdominal, articular, renal and mixed types based on the affected system. The disease is prevalent among children and extremely rare among adults. The majority of recent clinical studies have focused on children, while fewer studies have been conducted on adults. Isolated intussusception and renal failure are rather common in previous patients, but cardiac involvement is extremely rare.^[3]^ However, it is extremely rare for patients to develop multiple serious complications simultaneously. We report a case of an elderly patient with HSP who developed intussusception, renal failure and cardiac involvement sequentially.

## 2. Case report

A 72-year-old woman was admitted to the hospital with bilateral lower limb edema for 7 days. The patient previously had hypertension and diabetes mellitus, and she experienced recurrent bilateral lower limb edema and generalized a recurrent cutaneous rash without receiving comprehensive systemic treatment in the last 2 years. The patient’s vital signs were within normal limits, with a blood pressure of 140/80 mm Hg. The initial physical examination revealed bilateral lower extremity edema, particularly the right ankle, and skin pigmentation. Laboratory examinations revealed blood counts, C-reactive protein, creatinine kinase, albumin, prothrombin time, partial thromboplastin time, serum biochemistry assays, immunoglobulin, and complement levels were all within the normal range. The chest and abdominal computed tomography scans revealed no notable abnormalities. Urine routine analysis was positive for occult blood.

**Figure 1.**
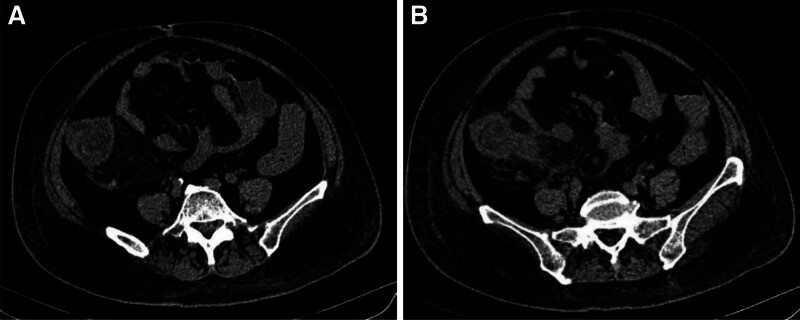
Segmental thickening of the intestinal wall and “concentric circles” in the ileocecal part (A, B).

**Figure 2.**
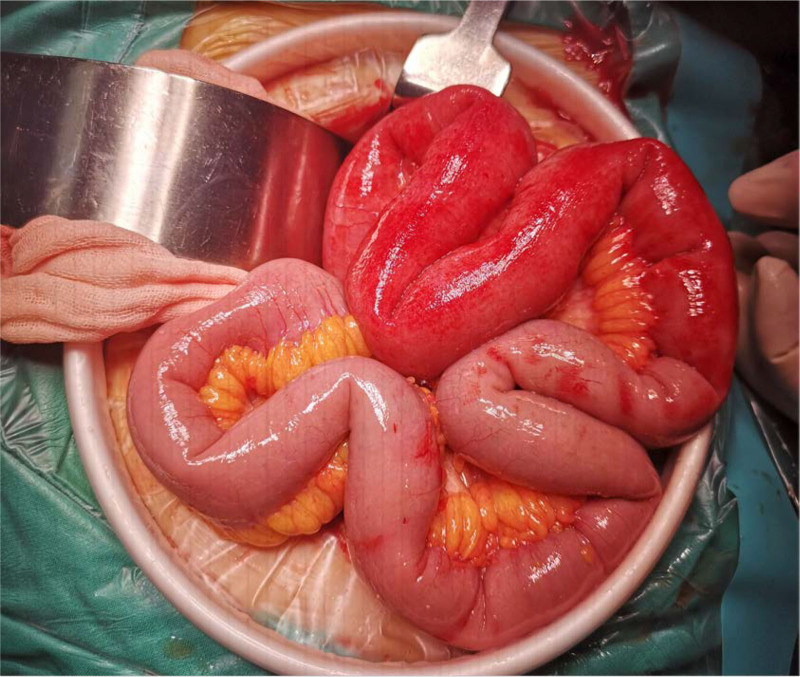
The intestinal mucosa is congested and edematous, while the intestinal wall is severely edematous and hemorrhagic.

**Figure 3.**
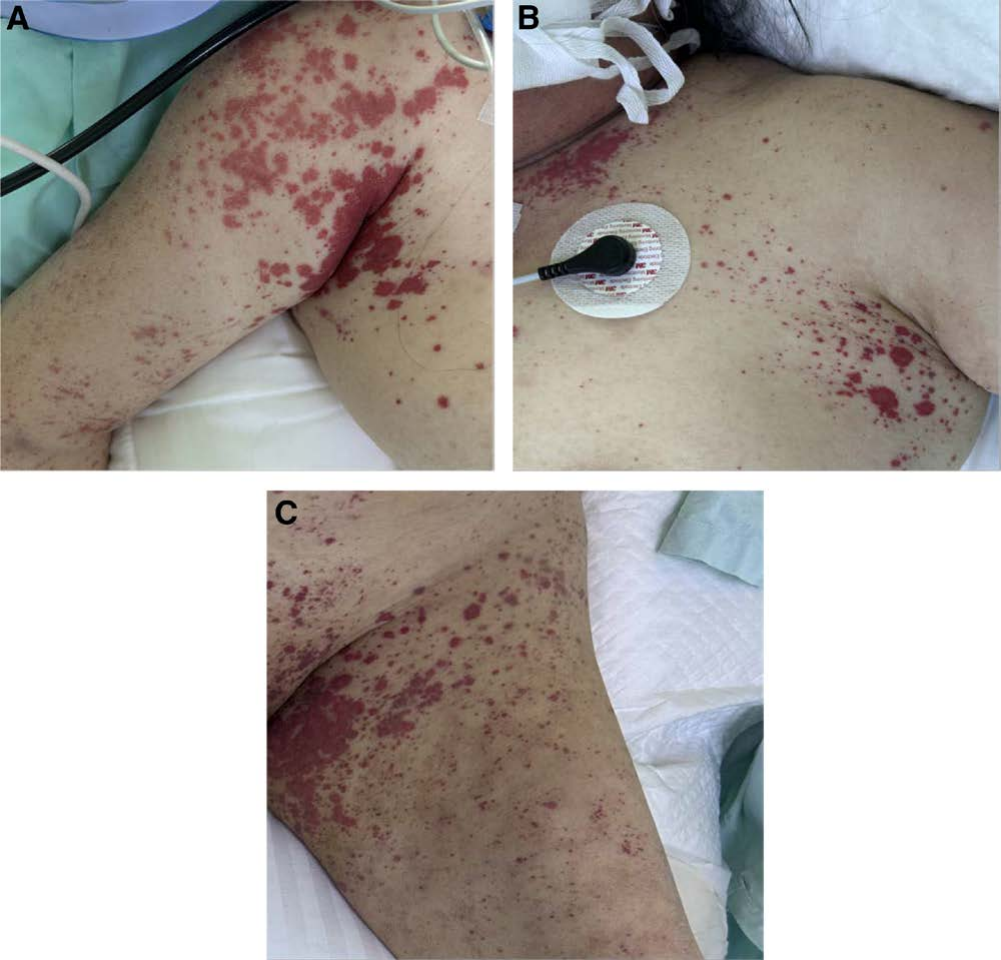
The patient presents with purpuric rash of anterior chest, upper limbs (A, B) and legs (C).

On the 7th day of admission, urine routine analysis was positive for occult blood and urine protein. Serum biochemical tests revealed high creatinine levels, and the patient had abnormal kidney function. On the 8th day of admission, purpura emerged on the lower limbs and back. We identified the patient with HSP and administered vitamin C and other symptomatic treatments. On the 12th day of admission, the patient complained of abdominal pain, so we urgently reviewed the abdominal computed tomography. It showed segmental thickening of the intestinal wall and “concentric circles” in the ileocecal part (Fig. 1A and B), so we diagnosed intussusception. The patient’s conservative treatment was ineffective, so she underwent emergency surgery. During the operation, the ileum was lodged in the cecum to cause the intussusception. The intestinal mucosa is congested and edematous, while the intestinal wall is severely edematous and hemorrhagic (Fig. 2). On the first postoperative day, the patient exhibited widespread purpura throughout the body (Fig. 3A–C); therefore, we administered glucocorticoids for symptomatic treatment. Unfortunately, the patient’s urine output gradually decreased, there was depressed edema of the lower limbs and renal function gradually deteriorated. Eventually, the patient developed acute renal failure. On the 6th surgical day, the patient underwent bedside hemofiltration and hemoperfusion therapies. After treatment, the patient’s edema gradually resolved, and her condition showed improvement. However, on the 9th postoperative day, the patient suddenly developed symptoms of heart failure, coughed up an amount of pink foamy sputum, with extensive wet rales in both lungs, blood pressure dropped. Two hours later, the electrocardiographic monitoring revealed ventricular escape rhythm, we immediately resuscitated the patient, and the patient resumed sinus rhythm after 5 minutes of cardiopulmonary resuscitation. Laboratory tests revealed higher levels of troponin, myoglobin and creatine kinase isoenzymes. The repeat transthoracic echocardiogram showed new-onset severe left ventricular (LV) systolic dysfunction with an LV ejection fraction of 38% (LV ejection fraction was 58% on admission). We considered myocardial involvement as the culprit. However, her family abandoned treatment, and the patient died.

## 3. Discussion

HSP is a vasculitis caused mainly by IgA deposition in the vascular wall, mainly involving small blood vessels and capillaries, which is characterized by nonthrombocytopenic palpable purpura of the skin. It frequently involves the skin, kidneys, joints and gastrointestinal tract.^[1]^ The etiology and pathogenesis of HSP are still unknown, and current studies prefer that HSP is an immune-responsive disease involving multiple inflammatory cells, cytokines, adhesion molecules, and oxidative stress mediated by IgA.^[4]^ The disease is common in children but extremely rare in adults, with an incidence of only 1.3 per 100,000. The course of the disease is often self-limiting and involves multiple systems, with severe renal insufficiency and even end-stage renal disease occurring in some patients, and complications are more severe in adults than in children.^[5]^ Among gastrointestinal symptoms, the most prevalent is abdominal pain, which might be induced by complications such as vasculitis, intestinal wall edema, intestinal wall hemorrhage, or intussusception and intestinal perforation, with the majority of the pain centering around the umbilicus. Nonetheless, the complication of intussusception is uncommon and more rare in adults.^[6]^ Intussusception in adults should be treated as soon as possible because if it is not detected in time, it can easily lead to intestinal necrosis and infectious shock in patients.^[7]^ In this case, the diagnosis of intussusception was clear, and we performed surgery urgently. In addition, significant cardiac complications in HSP are extremely rare. Thirteen patients have been confirmed to have cardiac complications in the past, and only 6 of them were confirmed by cardiac biopsy, of which 4 were found by autopsy. When patients develop cardiac problems, the majority of them become hemodynamically unstable, and the cardiac biopsy puts them at significant risk of death. Cardiac involvement is now largely detected by clinical signs such as congestive heart failure, atrioventricular block, escape rhythm, cardiac arrest, left ventricular systolic dysfunction, and elevated myocardial markers.^[8]^ In our case, myocardial markers and echocardiogram were normal at the time of admission, but during the treatment period, the patient developed symptoms of heart failure, ventricular escape rhythm, elevated myocardial markers and left ventricular systolic dysfunction, and the patient’s clinical presentation was consistent with the manifestations of previous occurrences of cardiac involvement. Therefore, we considered patients with cardiac complications.

Currently, the diagnosis of HSP is based on the 2006 classification of vasculitis in children by the European League Against Rheumatism and the European Society of Pediatric Rheumatology, with a palpable rash as a necessary condition. Other conditions include: (1) diffuse abdominal pain; (2) IgA deposits on biopsy at any site; (3) arthritis or arthralgia; (4) renal impairment, hematuria and/or proteinuria.^[9]^ Diagnosis is made by meeting the necessary conditions and any one of the other conditions. Additionally, while all patients with HSP present with a rash, there will be 10% of cases in which the rash appears within 2 weeks of the onset of manifestations in the abdomen, joints, or kidneys. Of the remaining 90% of cases that present with a rash, there will also be 10% to 15% in which the rash is initially unremarkable (such as limited to the buttock).^[10]^ In this case, the patient had no obvious rash early on, with subcutaneous edema as the main manifestation. It has been shown that half of current cases of HSP present with localized non-subcutaneous edema, especially in dorsal regions such as hands, ankles, and feet. Edema is uncommon elsewhere, such as on face, scalp, anterior scrotal area, labia, and lumbar spine.^[11–14]^ Whether subcutaneous edema can provide help in diagnosing HSP needs to be verified by future large-scale clinical studies.

The treatment of HSP is primarily symptomatic. The use of glucocorticoids in HSP has been a topic of controversy for many years. Some studies have indicated that early administration of glucocorticoid therapy can reduce the duration of abdominal pain and minimize gastrointestinal complications.^[15,16]^ Similarly, in patients with severe renal impairment, early application of gastrointestinal can significantly improve their symptoms.^[15–17]^ Hemoperfusion has also been demonstrated to effectively eliminate inflammatory mediators, regulate immune balance, and is considered a safe and effective method for treating severe HSP with gastrointestinal lesions.^[18]^ Furthermore, due to the low incidence but high mortality rate of HSP-related cardiac complications, there is no standardized treatment principle. In a recent study, rituximab was used and resulted in favorable therapeutic effects.^[8]^ We present a 72-year-old woman whose initial symptoms were unusual, and the progression of the disease is irregular. The patient developed early-stage renal and abdominal complications, we sequentially administered surgical intervention, glucocorticoids, hemofiltration and hemoperfusion, and her condition showed improvement. By the time cardiac complications develop, the optimal window of opportunity has passed. A study by Pillebout^[19]^ et al comparing the effects of high-dose prednisone alone with high-dose prednisone plus cyclophosphamide showed no difference between the 2 groups. In 2020, a meta-analysis including 20 studies, concluded that rituximab seems to be a safe and useful agent in HSP adult patients resistant or refractory to glucocorticoids or other immunosuppressive drugs.^[20]^ Therefore, early treatment with rituximab may improve the prognosis in adults with multisystem involvement. The treatment of adult-onset HSP is currently controversial. Most of the available studies have been conducted in pediatric populations, and the results have been extrapolated to adults. Numerous rigorously controlled clinical trials will be necessary in the future to elucidate the treatment of HSP in adult patients.

## 4. Conclusions

We present a unique case of an elderly patient with atypical symptoms and clinical progression in HSP and discuss the diagnosis and treatment of this patient. Early diagnosis and treatment of patients with atypical symptoms and critical conditions of HSP are essential. And we must adapt the treatment strategy in time based on the patient’s changing condition. Nevertheless, establishing an early diagnosis for patients exhibiting atypical clinical manifestations remains a significant challenge, compounded by the absence of definitive clinical guidelines for treatment.

## Author contributions

**Conceptualization:** Song Na.

**Data curation:** Jinquan Xu.

**Investigation:** Song Na, Lei Zhang, Luxin Kou.

**Resources:** Luxin Kou.

**Supervision:** Lei Zhang, Li Gang.

**Writing – original draft:** Song Na, Li Gang.

**Writing – review & editing:** Song Na.
